# Modulation of Protease Activated Receptor 1 Influences Human Metapneumovirus Disease Severity in a Mouse Model

**DOI:** 10.1371/journal.pone.0072529

**Published:** 2013-08-28

**Authors:** Laetitia Aerts, Marie-Ève Hamelin, Chantal Rhéaume, Sophie Lavigne, Christian Couture, WooJin Kim, Delia Susan-Resiga, Annik Prat, Nabil G. Seidah, Nathalie Vergnolle, Beatrice Riteau, Guy Boivin

**Affiliations:** 1 Centre de Recherche en Infectiologie du Centre Hospitalier Universitaire de Québec and Université Laval, Quebec, Canada; 2 Department of Anatomo-pathology, Institut Universitaire de Cardiologie et de Pneumologie de Québec, Université Laval, Quebec, Canada; 3 Laboratory of Biochemical Neuroendocrinology, Clinical Research Institute of Montreal, Montreal, Canada; 4 Institut National de la Santé et de la Recherche Médicale, Centre National de la Recherche Scientifique, Université de Toulouse, Université Paul Sabatier, Centre de Physiopathologie de Toulouse Purpan, Toulouse, France; 5 Department of Physiology and Pharmacology, University of Calgary, Alberta, Canada; 6 Virologie et Pathologie Humaine, Université Lyon1, Faculté de Médecine RTH Laennec, Lyon, France; 7 Centre de Tours-Nouzilly Institut National de la Recherche Agronomique, Nouzilly, France; Louisiana State University, United States of America

## Abstract

Human metapneumovirus (hMPV) infection causes acute respiratory tract infections (RTI) which can result in hospitalization of both children and adults. To date, no antiviral or vaccine is available for this common viral infection. Immunomodulators could represent an interesting strategy for the treatment of severe viral infection. Recently, the role of protease-activated receptors (PAR) in inflammation, coagulation and infection processes has been of growing interest. Herein, the effects of a PAR1 agonist and a PAR1 antagonist on hMPV infection were investigated in BALB/c mice. Intranasal administration of the PAR1 agonist resulted in increased weight loss and mortality of infected mice. Conversely, the PAR1 antagonist was beneficial to hMPV infection by decreasing weight loss and clinical signs and by significantly reducing pulmonary inflammation, pro-inflammatory cytokine levels (including IL-6, KC and MCP-1) and recruitment of immune cells to the lungs. In addition, a significant reduction in pulmonary viral titers was also observed in the lungs of PAR1 antagonist-treated mice. Despite no apparent direct effect on virus replication during *in vitro* experiments, an important role for PAR1 in the regulation of furin expression in the lungs was shown for the first time. Further experiments indicated that the hMPV fusion protein can be cleaved by furin thus suggesting that PAR1 could have an effect on viral infectivity in addition to its immunomodulatory properties. Thus, inhibition of PAR1 by selected antagonists could represent an interesting strategy for decreasing the severity of paramyxovirus infections.

## Introduction

Human metapneumovirus (hMPV) is a paramyxovirus responsible for acute respiratory tract infections that are especially severe in young children [1,2], elderly people [3,4] and immunocompromized individuals [5,6]. Limited reports in humans [3,7,8] and extensive studies in animals [9–13] have shown that severe hMPV infection is associated with the disruption of the epithelial architecture and the presence of inflammatory infiltrates around and within alveoli. Among the diverse small animal models that have been evaluated, BALB/c mice have emerged as an excellent model for recapitulating hMPV infection, including efficient viral replication in the lungs, concomitant pulmonary inflammation and airway obstruction as well as disease severity that correlates with increasing viral inoculums [10,14–16]. BALB/c mouse studies have also shown that the immune response to hMPV infection can be both disproportionate and inefficient, characterized by a weak innate response and a Th2-biased adaptive immune response, leading to impaired virus clearance and prolonged pulmonary inflammation [10,14–16].

To date, no vaccine or specific antiviral agents are available for the treatment of hMPV infection [17]. Ribavirin, a nucleoside analogue licensed for treatment of severe human respiratory syncytial virus (hRSV) infections, exhibits moderate *in vitro* activity against hMPV and was found to be beneficial in the mouse model [18,19]. Indeed, this antiviral has been used to treat a few severe cases of hMPV pneumonia in immunocompromised individuals [20–22]. The mechanisms of action of ribavirin do not appear to be limited to the disruption of the viral purin metabolism and inhibition of viral RNA polymerase. It has been shown that this drug could also upregulate IL-2, TNF-α, interferon-γ and downregulate Th2 cytokines such as IL-10 *in vitro*, suggesting an immunomodulatory as well as an antiviral effect [23]. However, ribavirin is associated with many side-effects and teratogenicity [24], which limits its widespread use. Thus, the development of new antivirals and/or immunomodulatory strategies is warranted for the treatment of severe hMPV infections.

Secretory serine proteases have long been known to play an important role in several physiological processes such as hemostasis (primary hemostasis, coagulation and fibrinolysis) and immune responses to infection [25]. Increasing interactions between these two systems are being discovered, including during viral infection [26]. Serine proteases of the coagulation cascade, such as thrombin, are able to activate complement components [27,28] and to regulate cell function through the activation of a family of G-protein coupled receptors called protease-activated receptors (PAR) [29–33]. PARs have a unique mechanism of activation in which irreversible proteolytic cleavage within the extracellular N-terminus of the receptor exposes a new N-terminal sequence that acts as a tethered ligand. The latter interacts with the second extracellular loop of the receptor, hereby activating the receptor [30,34,35]. Four PARs have been characterized until now (PAR1, PAR2, PAR3 and PAR4). They are all activated by thrombin except PAR2 for which the main activating protease is trypsin [30,35].

PAR1 in particular is a ubiquitous receptor present in platelets, endothelial and epithelial cells, neurons, fibroblasts, smooth muscle cells, leukocytes and tumor cell lines [30]. PAR1 is a receptor involved in many physiological processes including the cardiovascular, respiratory and central nervous systems as well as in embryogenesis, cancer and inflammation [35]. In fact, some PAR1 antagonists are currently in clinical trials for their antiplatelet activities [36,37]. The role of PAR1 in the pathogenesis of infectious diseases has recently been investigated for a number of pathogens including feline immunodeficiency virus [38], *Mycobacterium tuberculosis* [39], dengue virus [40], herpes simplex virus [41,42] and influenza A virus [43,44]. For the latter pathogen, upregulation of PAR1 was observed in the airways of mice infected with the highly pathogenic influenza A/PR-8/34 (H1N1) virus [43]. Based on these observations, we hypothesized that PAR1 could also play an important role in modulating the immune response during severe hMPV infection. Therefore, we treated hMPV-infected BALB/c mice with either a selective PAR1 agonist (TFLLR-NH_2_) or a PAR1 antagonist (SCH79797) and then evaluated disease severity. We observed that PAR1 activation increased weight loss and decreased survival in our hMPV mouse model. In contrast, administration of a PAR1 antagonist decreases disease severity and significantly reduced lung cytokines and inflammation in BALB/c mice. The protective effect of the PAR1 antagonist could also be explained by a decrease in furin-mediated cleavage of the viral fusion (F) protein.

## Results

### PAR1 agonist (TFLLR-NH_2_) and PAR1 antagonist (SCH79797) do not exhibit antiviral activity against hMPV in LLC-MK2 cells

We sought to investigate whether the PAR1 agonist or antagonist had an effect on viral replication *in vitro*. In a first experiment, uninfected LLC-MK2 monolayers were pre-incubated with the compounds and then infected. However, cell monolayers detached at concentrations > 0.8 µM of the PAR1 antagonist (SCH79797), possibly due to the pro-apoptotic effect of this reagent [45] and the toxicity of the vehicle (DMSO). No toxicity was observed using concentrations up to 250 µM of the PAR1 agonist (TFLLR-NH_2_). Using concentrations of up to 0.8 µM for the antagonist and 250 µM for the agonist, no antiviral effect was observed. In a second experiment, hMPV was pre-incubated with the compounds (maximum concentration of 0.8 µM) before addition to LLC-MK2 monolayers. No antiviral effect was again observed when the compounds were removed and replaced by fresh medium after the adsorption phase or when the compounds were replenished daily throughout the experiment (not shown).

### PAR1 agonist and antagonist have an inverse dose-dependent effect on hMPV infection in vivo

**Figure 1:**
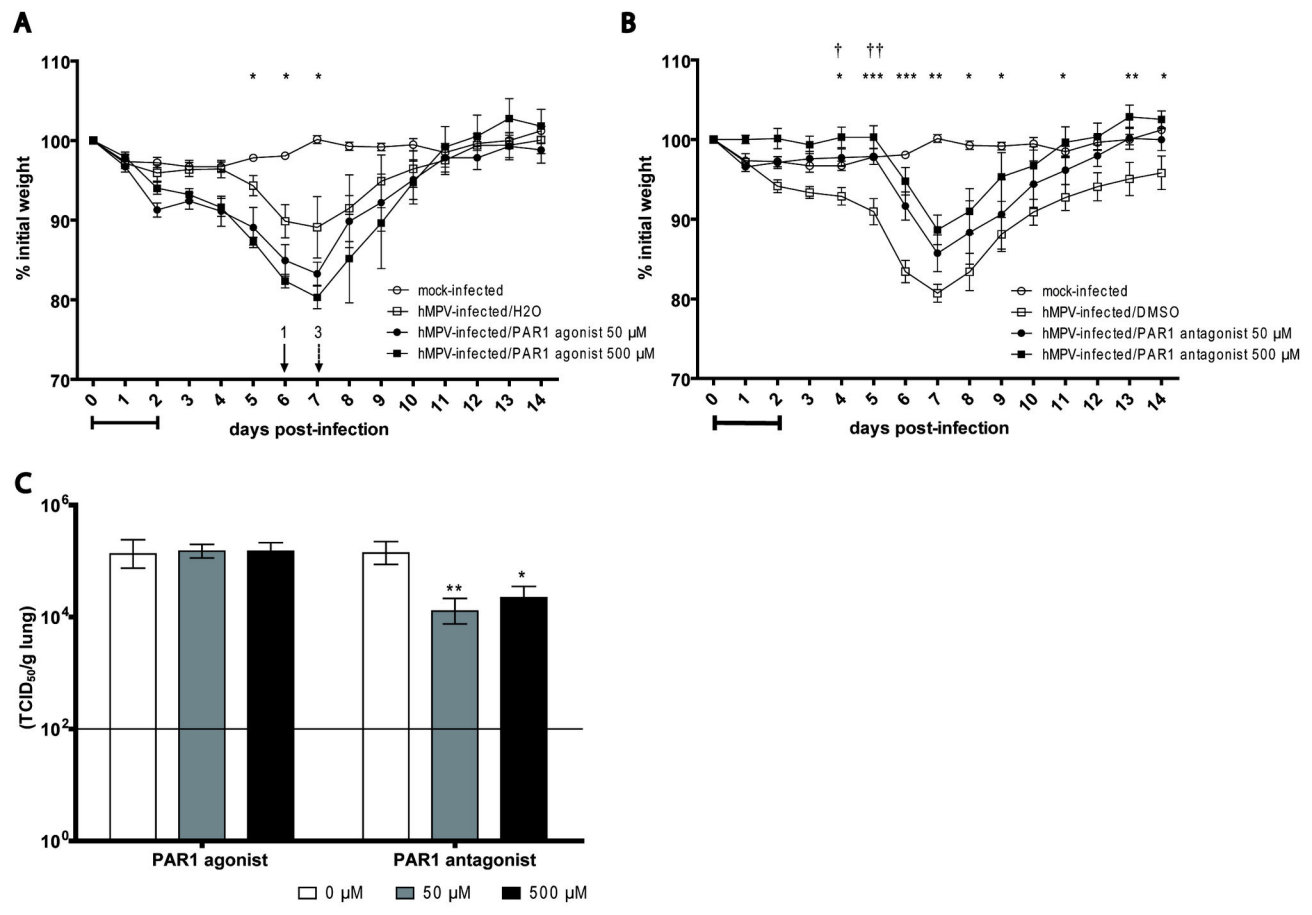
PAR1 agonist or antagonist dose-dependent effect on hMPV infection during a 3-day treatment in mice. Groups of 12 mice were infected intranasally with hMPV (4-6 x10^5^ TCID_50_) or mock infected and simultaneously treated for 3 days with a single daily dose of 50 or 500 µM of PAR1 agonist (TFLLR-NH_2_), PAR1 antagonist (SCH79797) or their respective vehicles. A) and B) Weight loss and mortality were monitored daily for 14 days for mice treated with the PAR1 agonist and antagonist, respectively. The horizontal bar underneath the graphic indicates the timing and duration of treatment. Arrows and numbers indicate the mice that reached the endpoint and were sacrificed (full line: mice treated with 50 µM of PAR1 agonist, dotted line: mice treated with 500 µM of PAR1 agonist). Significant differences in weight loss were observed between mice treated with 50 µM (†) or 500 µM (*) of compound compared to untreated mice based on a two-way ANOVA (* p<0.05, ** p<0.01, *** p<0.001, † p<0.05, † † p<0.01). C) Viral titers were determined by TCID_50_ in lung homogenates at day 5 pi. Significant differences were observed between treated or untreated mice as determined by one-way ANOVA. (* p<0.05, ** p<0.01) The bar indicates the lower limit of detection.

In a first *in vivo* experiment, BALB/c mice were infected intranasally with hMPV (4-6x10^5^ TCID_50_) or mock infected and simultaneously treated intranasally, for a period of 3 days, with a single daily dose of 50 or 500 µM of PAR1 agonist (TFLLR-NH_2_) or PAR1 antagonist (SCH79797). PAR1 agonist- or PAR1 antagonist-treatment of uninfected mice did not induce weight loss, mortality or any signs of toxicity (data not shown). Mortality was only observed in PAR1-agonist treated mice (17% on day 6 post infection (pi) and 50% on day 7 pi for mice treated with 50 µM and 500 µM of PAR1 agonist, respectively). These groups also had a greater weight loss compared to infected, vehicle-treated mice (Figure 1A). Conversely, weight loss was significantly reduced in a dose-dependent manner in PAR1 antagonist-treated mice compared to the infected, vehicle-treated group (Figure 1B). No significant difference in pulmonary viral titers was observed between PAR1 agonist-treated mice and vehicle-treated controls. In contrast, viral titers in the lungs of PAR1 antagonist-treated mice were significantly lower than those of vehicle-treated mice by about 1 log (Figure 1C). Thus, we conclude that PAR1 plays a deleterious role in the pathogenesis of hMPV infections.

### Prolonged treatment with the PAR1 antagonist (SCH79797) prevents symptomatic hMPV infection

**Figure 2:**
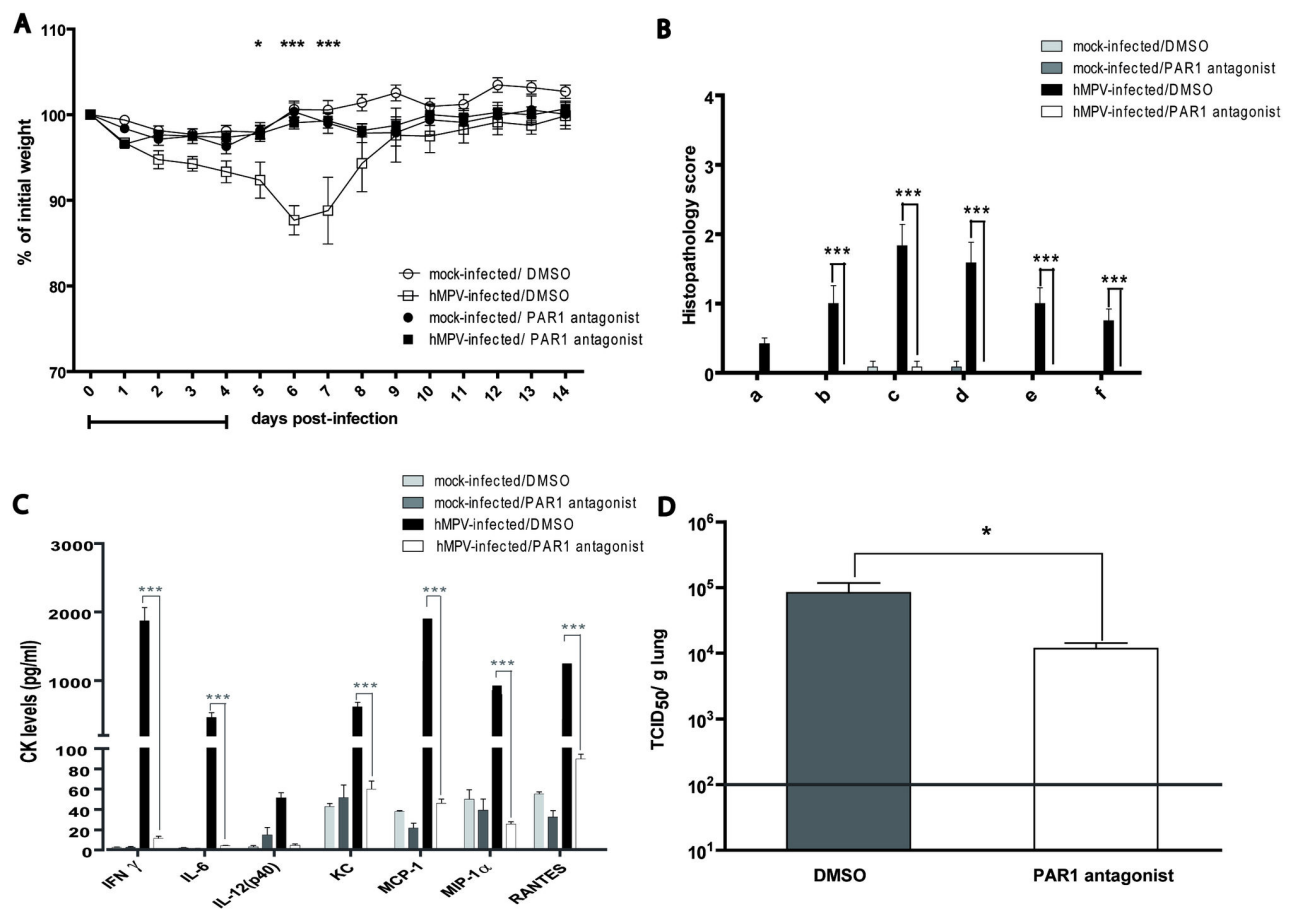
Effect of PAR1 antagonist on hMPV infection during a 5-day treatment in mice. Groups of 18 mice were infected intranasally with hMPV (6-8 x10^5^ TCID_50_) or mock infected and simultaneously treated for 5 days with a single daily dose of 500 µM of PAR1 antagonist (SCH79797) or with their respective vehicle. A) Weight loss and mortality were monitored on a daily basis for 14 days. The horizontal bar underneath the graphic indicates the timing and duration of treatment. * indicate a significant difference between PAR1 antagonist (SCH79797) treated mice and untreated controls based on a two-way ANOVA (* p<0.05, *** p<0.001). B) On day 5 pi, 6 mice per group were euthanized and lungs were removed, fixed, embedded, sectioned and stained for histopathology. The degree of inflammation was determined using a semi-quantitative scale (a: bronchial/endo-bronchial inflammation; b: peribronchial inflammation; c: perivascular inflammation; d: interstitial inflammation; e: pleural inflammation; f: intra-alveolar inflammation). Significant differences in pulmonary histopathological scores were observed between treated and untreated mice based on a two-way ANOVA(*** p<0.001). C) Pulmonary cytokine/chemokine levels in lung homogenates were determined by Luminex (IFNγ, IL-4, IL-6, IL-12(p40), IL-12(p70), KC, MCP-1, MIP-1α and RANTES). IL-4 and IL-12(p70) data were removed from the graph since no detectable values were obtained for these cytokines in any of the groups. Significant differences in pulmonary pro-inflammatory cytokine/chemokine levels were observed between treated and untreated mice based on a two-way ANOVA (*** p<0.001). D) Viral titers were determined in lung homogenates by TCID50. Significant differences in pulmonary viral titers were observed between PAR1 antagonist-treated mice and infected untreated mice based on a Student t-test. (* p<0.05).

In the preliminary PAR1 experiment, we observed that a 3-day treatment with the PAR1 antagonist (SCH79797) reduced weight loss in a dose-dependent manner. In order to evaluate if a longer treatment had a more pronounced effect, the compound was administered as a single daily dose of 500 µM for 5 days, starting at the time of infection. PAR1 antagonist-treatment of uninfected mice did not induce weight loss, mortality or any signs of toxicity. PAR1 antagonist-treated and infected mice remained asymptomatic i.e. showed no weight loss, reduced activity or ruffled fur throughout the experiment (Figure 2A). These findings were confirmed in a second experiment using 18 mice per group (data not shown). Moreover, on day 5 pi, a significant decrease in pulmonary inflammation was observed in treated mice (Figure 2B) that correlated with a significant decrease in pro-inflammatory cytokine levels (Figure 2C). In addition, viral replication in the lungs on day 5 pi, was significantly reduced by about 1 log in PAR1 antagonist-treated mice, compared to controls, confirming the results of the previous 3-day experiment (Figure 2D). Thus, we conclude that blocking PAR1 protects mice from symptomatic hMPV disease.

### Cell recruitment in lungs of mice treated with the PAR1 antagonist

**Figure 3:**
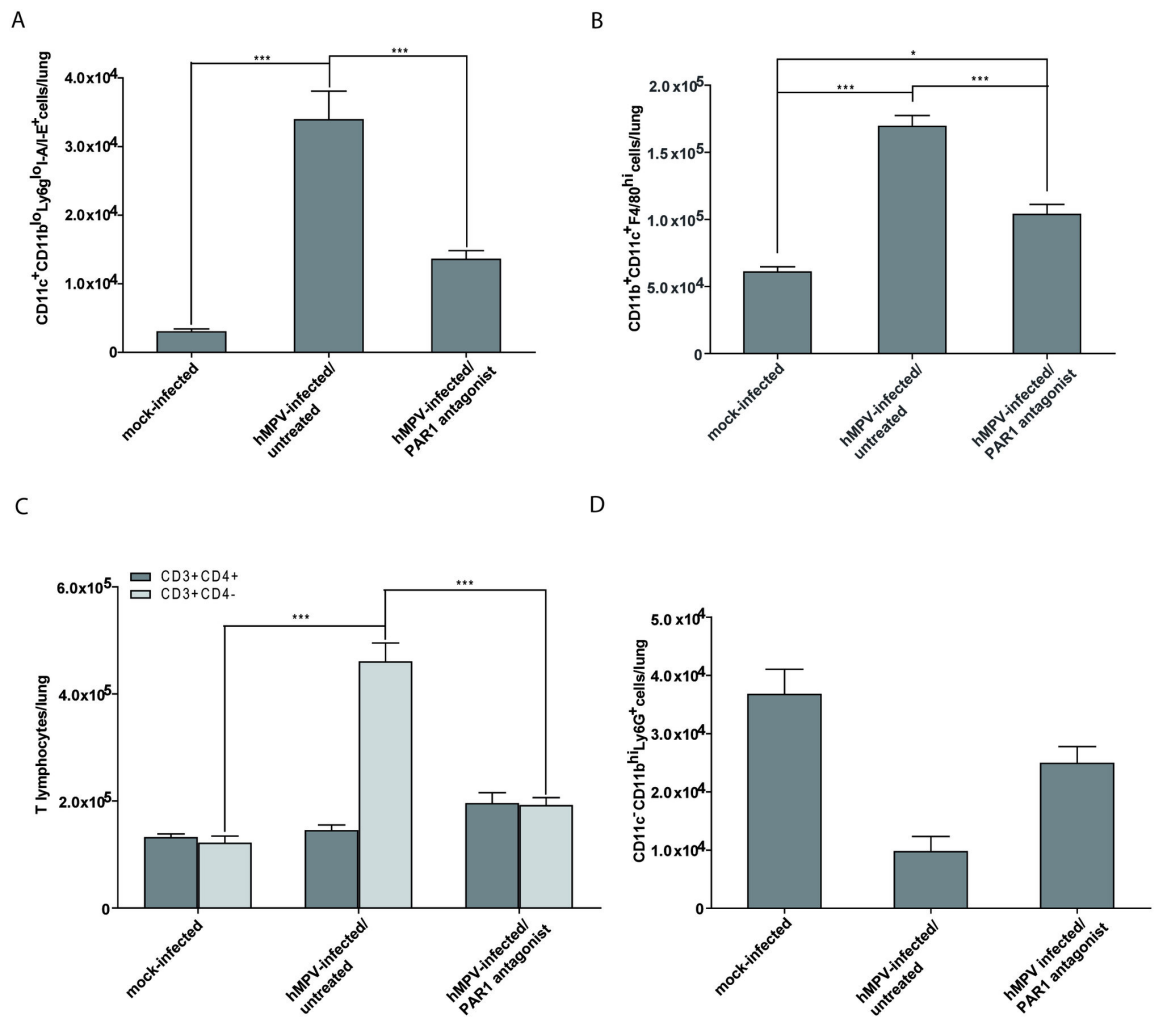
Cell recruitment in the lungs of hMPV-infected mice treated with the PAR1 antagonist. Groups of 6 mice were infected intranasally with hMPV (7 x10^5^ TCID_50_) or mock infected and simultaneously treated for 5 days with a single daily dose of 500 µM of PAR1 antagonist (SCH79797). Mice were sacrificed on day 5 pi, lungs were removed, homogenized in HBSS and analyzed by flow cytometry for the presence of (A) dendritic cells expressing the MHC II molecules I-A/I-E (CD11c^+^CD11b^lo^Ly6G ^lo^I-A/I-E^+^), (B) macrophages (CD11c^+^CD11b^+^F4/80^hi^), (C) T lymphocytes (CD3^+^CD4^+^ or CD3^+^CD4^-^) and (D) neutrophils (CD11c^-^CD11b^hi^Ly6G^+^). Significant differences in recruited cells were observed between treated and untreated mice based on a one-way ANOVA. (* p<0.05, *** p<0.001).

Immune cell populations present in the lungs on day 5 pi were analyzed by flow cytometry in mice treated for 5 days starting at the time of infection with 500 µM of the PAR1 antagonist (Figure 3). The immune cell populations analyzed included activated dendritic cells (CD11c^+^CD11b^lo^Ly6g ^lo^I-A/I-E^+^), macrophages (CD11b^+^CD11c^+^F4/80^hi^), T lymphocytes (CD3^+^CD4^+^ or CD3^+^CD4^-^) and neutrophils (CD11c^-^CD11b^hi^Ly6G^+^). Significantly reduced populations of activated dendritic cells, macrophages and CD4^-^ T-lymphocytes were observed in the lungs of PAR1 antagonist-treated mice compared to hMPV-infected untreated mice. Although there appears to be a decrease in neutrophils in infected untreated mice, CD11c-CD11bhiLy6G+ events were extremely low. This population no longer followed a Gaussian distribution and we are therefore reluctant to draw any definite conclusion for this specific population. These results confirm that the PAR1 antagonist protects mice from potentially deleterious lung inflammation induced by hMPV infection.

### PAR1 antagonist (SCH79797) treatment decreases furin expression in the lungs of hMPV-infected mice

**Figure 4:**
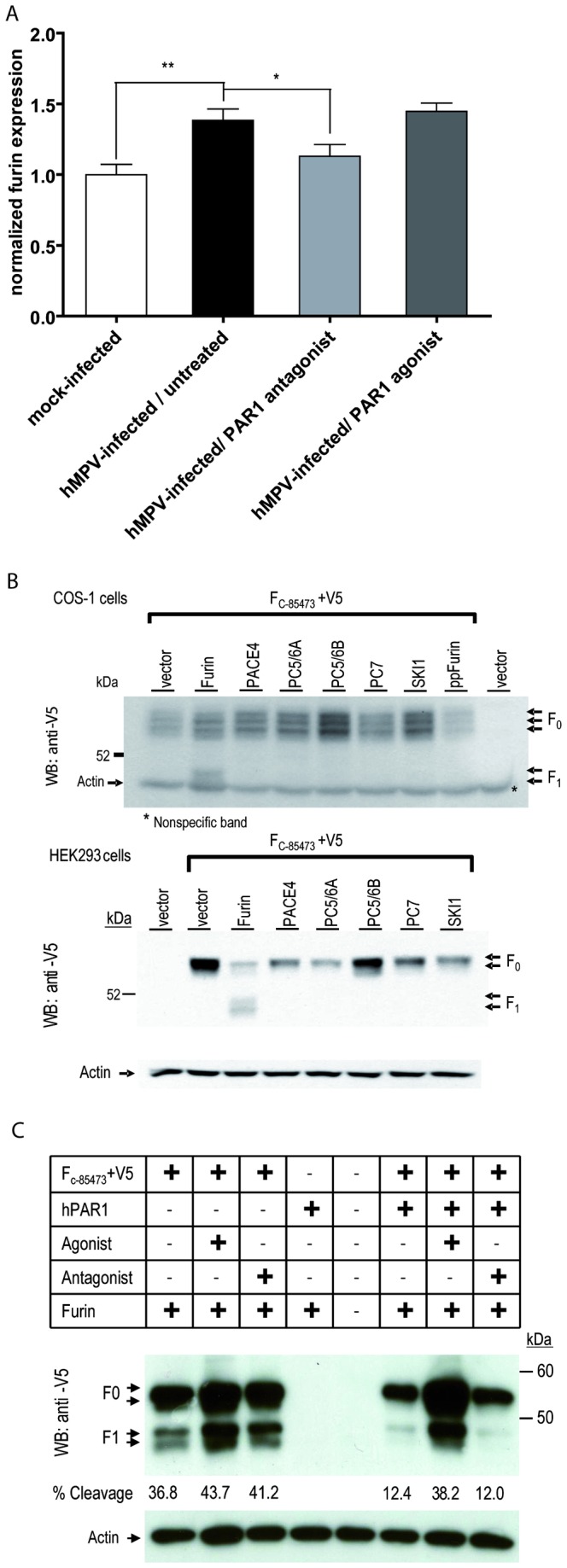
Effect of PAR1 agonist or antagonist treatment of hMPV-infected mice on furin expression. A) Groups of 6 mice were infected intranasally with hMPV (7x10^5^ TCID_50_) or mock infected and simultaneously treated for 5 days with a single daily dose of 500 µM of PAR1 agonist (TFLLR-NH_2_) or PAR1 antagonist (SCH79797). Mice were sacrificed on day 5 pi, lungs were removed and snap frozen. RNA was extracted and furin transcript levels were determined using RT-PCR. Significant differences in furin transcript levels were observed between treated and untreated mice based on a Student T-test (* p=0.05, ** p<0.01) B) COS-1 and HEK293 cells were co-transfected with a plasmid encoding the hMPV F protein containing a V5-tag and a plasmid encoding one of the proprotein convertases. Cell lysates were analyzed by western blot using a V5 mAb. Furin is the only convertase capable of cleaving the full length precursor protein (F0) into its activated form, resulting in the shorter C-terminal subunit (F1). C) Recombinant human PAR1 and furin are co-tranfected with the hMPV F protein containing a V5-tag in HEK293 cells with/without hPAR1 agonist, (100 μM) or antagonist (0.1 μM). Western blot analysis of cell lysates using an anti-V5 mAb is shown.

The proprotein convertases, especially furin, have been shown to process a number of cell surface glycoproteins of infectious viruses both at single and paired basic residues. The minimal cleavage site is RXXR **↓**, exhibiting a P1 and P4 Arg residues [46]. In order to tentatively investigate the possible mechanism by which PAR1 compounds can influence viral replication, we analyzed furin transcripts in the lungs of hMPV-infected animals by RT-PCR. PAR1 compounds were administered for 5 days at a dose of 500 µM, starting at the time of infection, and lungs were harvested on day 5 pi. HMPV infection resulted in a significant increase in furin transcripts (by 38%) in mouse lungs compared to uninfected mice (Fig. 4A). Treatment of infected mice with the PAR1 agonist did not significantly alter furin expression. However, treatment of infected mice with the PAR1 antagonist significantly reduced furin transcript levels (by 18%) compared to hMPV-infected/untreated mice. Using a pIRES vector expressing each proprotein convertase, we previously showed a similar expression of each convertase and their ability to cleave selected substrates [47,48]. Thus, we used these same constructs to show that furin was the only tested proprotein convertase that was able to cleave the hMPV fusion precursor protein into its active form (Figure 4B) in two cell lines (COS-1 and HEK293). We then investigated the effect of PAR1 on the cleavage of the hMPV fusion precursor protein (Figure 4C) in this *in vitro* cleavage assay. Treatment of F protein/furin co-transfected HEK293 cells with either the PAR1 agonist or antagonist did not alter F cleavage (43.7 and 41.2% cleavage, respectively, compared to 36.8%). However, co-transfection of cells with cDNA encoding the F protein, furin as well as recombinant human PAR1 (rhPAR1) reduced F cleavage from 36.8% to 12.4%. When these rhPAR1 expressing co-transfected cells were treated with the PAR1 antagonist, F protein cleavage remained low (12.0%), whereas PAR1 agonist treatment restored F protein cleavage to the basal level observed in cells without rhPAR1 expression (38.2%), confirming a role for PAR1 in the cleavage of the hMPV fusion protein.

## Discussion

In this study, we showed that PAR1 activation increases weight loss and mortality in a mouse model of hMPV infection. Conversely, an antagonist of PAR1 (SCH79797) prevents clinical signs and reduces lung inflammation and viral titers, when administered at the time of viral infection. The reduction in lung viral titers by the PAR1 antagonist was probably not due to a direct antiviral effect but was more likely mediated through a reduction in furin expression and/or activity and viral infectivity.

hMPV infection causes a wide spectrum of diseases from mild upper respiratory tract infections (RTI) to severe lower RTI such as bronchiolitis or pneumonia and can result in hospitalization of both children and adults [49]. HMPV is one of the most prominent respiratory pathogens in children, accounting for 5–15% of pediatric hospitalizations for respiratory tract infections [49,50]. A recent study estimated the hospitalization rate for hMPV in older adults to be 9.8 per 10000 residents, a rate similar to those of hRSV and influenza [51]. Hospitalized patients are currently treated symptomatically, since no specific prophylactic or therapeutic modality is available. Although not approved for this indication, ribavirin has been administered as an antiviral agent in a few severe cases of hMPV pneumonia with variable outcomes [20–22]. Several hMPV vaccines have been investigated in animal models, including live-attenuated and subunit vaccines; however, vaccine-induced immunity was often partially protective and/or waned rapidly [52]. Importantly, hMPV-inactivated vaccines have been shown to induce an enhanced disease upon infection reminiscent of hRSV-inactivated vaccines [52–54]. Clearly, alternative approaches for the management of hMPV infections are needed.

We used an agonist (TFLLR-NH_2_) and an antagonist (SCH79797) of PAR1 to investigate the role of this receptor during hMPV infection. To further investigate the PAR1 agonist specificity, we examined its well-known effect on intestinal permeability [55] in wild-type and PAR1-knockout mice. To this end, ^51^Cr-EDTA was infused in the colon of the mice and the effect of the PAR1 agonist on the passage of ^51^Cr-EDTA to the blood, reflecting intestinal permeability was measured (Figure S1A). As expected, mice treated with the PAR1 agonist peptide TFLLR displayed enhanced intestinal permeability, compared to saline control treated mice. In marked contrast, mouse treatment with the RLLFT inactive control peptide had no effect on permeability, arguing against potential nonspecific effects of the agonist. More importantly, the effect of the agonist on increased intestinal permeability was abolished in PAR1-deficient mice. Altogether, these results show that the agonist specifically activates PAR1 in mice. Furthermore, this agonist peptide has been also used to specifically activate PAR1 in mice elsewhere [56–58]. Subsequently, we also investigated whether the PAR1 antagonist was capable of blocking the specific PAR1 signaling in vivo. We found that antagonist treatment prevented increased intestinal permeability associated with administration of the PAR1 agonist peptide to wild-type mice (Figure S1B). Thus, the effect of the antagonist is likely related to its ability to specifically block PAR1 *in vivo*. Furthermore, the antagonist has also been used to block PAR1 activation in rodent models elsewhere [56,58–60].

In our mouse model, inhibition of PAR1 activation using the antagonist SCH79797 was clearly beneficial by decreasing the severity of hMPV disease. Most strikingly, when treatment was started simultaneously with hMPV-infection and was continued for 5 days, no weight loss or clinical signs were observed (Figure 2). Evaluation of pulmonary inflammation and viral titers was performed on day 5 pi, the time point at which both parameters peak in untreated hMPV-infected mice [10]. During this acute phase of infection, pulmonary inflammation mostly consists of interstitial, perivascular and alveolar inflammation and all these parameters were significantly reduced by treatment with the PAR1 antagonist. In line with this observation, we also showed a significant reduction in the levels of key cytokines/chemokines that are usually increased in hMPV infection (including IFN-γ, IL-6, KC, MCP-1, MIP-1α and RANTES) [10]. Some of these cytokines namely IL-6, IL-8 (the human equivalent of murine KC) and MCP-1 have been previously shown to be up-regulated by PAR1 activation or down-regulated by PAR1 inhibition in non-infected human respiratory epithelial cells in models of asthma and idiopathic pulmonary fibrosis [61–64]. Furthermore, SCH79797 significantly reduced MCP-1 expression in *M. tuberculosis* H37Rv-infected cells *in vitro* [39]. Finally, Khoufache et al. recently demonstrated that SCH79797 treatment of influenza A-infected mice also significantly reduced IL-6, KC and RANTES levels in broncho-alveolar lavages [44].

We further investigated immune cell populations (activated dendritic cells, macrophages, T lymphocytes and neutrophils) in the lungs of hMPV-infected mice treated with the PAR1 antagonist on day 5 pi using flow cytometry. We did not observe significant differences in pulmonary neutrophils between any of the groups. However, the PAR1 antagonist treatment significantly reduced the recruitment of lung macrophages (Figure 3). PAR1 is expressed on both human and murine dendritic cells and its activation induces the release of MCP-1, IL-10, and IL-12 from these cells [65]. Moreover, PAR1 signaling on dendritic cells has been shown to sustain a lethal LPS-induced inflammatory response in mice [66]. In our study, we also observed a significant reduction in dendritic cells present in the lungs of PAR1 antagonist-treated mice compared to untreated hMPV-infected mice. Activation of PAR1 on human T cells induces the secretion of IL-6 [67] and may have an effect on T cell receptor signaling [68]. In addition, T cell migration to the liver of PAR1-deficient mice was reduced by 70% in a model of liver fibrosis, suggesting a role for PAR1 in T cell migration [69]. We did not observe a significant difference in CD4^+^ T lymphocytes between any of the groups, yet significantly less CD4^-^ T-lymphocytes were found in the lungs of PAR1 antagonist-treated mice. It remains to be confirmed, however, whether these CD4^-^ lymphocytes represent cytolytic T lymphocytes.

To provide further evidence that blocking PAR1 is protective during hMPV infections, we evaluated the effect of another PAR1 antagonist (SCH530348) on the outcome of hMPV infection in our mouse model. SCH79797 was used as a control (Figure S2). In both treated groups, hMPV-infected mice showed reduced weight loss although the reduction was more important with SCH79797. In fact, as was the case in the first 5-day experiment, the weight of SCH79797-treated mice remained similar to that of uninfected mice and no reduced activity or ruffled fur were observed. Treatment with either antagonist also reduced mortality induced by hMPV infections. Compared to 33% mortality rate for the untreated group, only 17% and 0% mortality rates were observed for the SCH530348- and SCH79797-treated groups, respectively. Thus, these results confirm that blocking PAR1 is protective during hMPV infections.

Immunomodulation, as a therapy, offers several advantages over conventional antiviral strategies. Because the host is the target of such a therapy, immunomodulation avoids the selective pressure on the pathogen that leads to the development of antimicrobial resistance. In addition, because of the non-specific nature of the innate immune system, its modulation could result in broad-spectrum protection against a wide range of microbial pathogens. The use of immunomodulators for viral infections has been most extensively investigated for chronic infections such as HIV and viral hepatitis infections and some of these agents like interferon are in clinical use [70]. With the advent of severe respiratory distress syndromes due to A/H5N1 avian influenza and SARS-coronavirus infections and in particular since the 2009 influenza pandemic, the interest for immunomodulators in acute viral infections has been increasing. Several anti-inflammatory therapies for influenza A infections have been evaluated so far, including sphingosine analogues, NF-κB inhibitors, antimicrobial peptides, COX-2 inhibitors and macrolides [71–73]. The latter are antibiotics with known anti-inflammatory and immunomodulatory properties that have shown promising results in several viral respiratory infections caused by influenza A, rhinovirus and hRSV [74]. Like macrolides, PAR1 antagonists may have a broad spectrum of activity. Indeed, Khoufache et al. recently reported results similar to ours in murine models of influenza A infections including those caused by A/H5N1 and oseltamivir-resistant A(H1N1) pdm09 strains [44]. In addition, unpublished data from our group indicates that PAR1 antagonists can significantly decrease hRSV lung titers and key pulmonary cytokines (IL-6, MCP-1, RANTES) in a similar mouse model highlighting the broad-spectrum potential of these compounds.

We found that PAR1 antagonist-treated and infected mice had reduced pulmonary viral titers on day 5 pi. This observation suggests a direct or indirect effect of PAR1 on viral replication. Such direct antiviral effect was not observed in our *in vitro* cell culture experiments, although testing was limited by cytotoxicity so that we cannot completely rule out a direct antiviral effect. On the other hand, viral entry into the host cell is mediated by the hMPV F surface glycoprotein. This protein is synthesized as an inactive precursor protein that requires proteolytic cleavage to become active [75]. The cleavage motif of the HMPV F protein at **R**SQ**R↓**FV contains a minimal furin cleavage site, i.e., **R**XX**R↓** present in many of its *in vivo* substrates [76,77]. Even though cleavage of the HMPV F protein requires the addition of trypsin *in vitro*, other enzymes could be involved in activating the F protein *in vivo*. Indeed, we found furin expression levels to be significantly increased in the lungs of hMPV-infected mice compared to mock-infected mice. In addition, using an *in vitro* cleavage assay, we clearly showed for the first time that, of all tested proprotein convertases, only furin was capable of cleaving the hMPV F precursor protein. Moreover, expression of recombinant human PAR1 (rhPAR1) reduced F protein cleavage in this assay; whereas treatment of rhPAR1 expressing cells with the PAR1 agonist restored F protein cleavage, treatment with the PAR1 antagonist did not. Since activation of PAR1 quickly initiates the internalization and degradation of the receptor [78] we hypothesize that the presence of unactivated PAR1 at the cell surface inhibits furin-mediated F protein cleavage. These *in vitro* observations could, at least partly, explain the slight reduction in viral titers observed in the lungs of hMPV-infected, PAR1 antagonist-treated mice. Considering that endogenous activators of PAR1 are present during *in vivo* infection, the inhibition of PAR1 activation with the PAR1 antagonist and the subsequent inhibition of PAR1 internalization and degradation could lead to reduced F cleavage and could therefore contribute to decreased hMPV infectivity. Taken together, these data suggest that furin levels and/or activity could have an effect on hMPV infectivity. On a similar note, PAR1 antagonists have been shown to decrease the plasmin-mediated cleavage of the influenza hemagglutinin protein with subsequent reduction in mouse lung viral titers [44]. Although PAR1 modulation of furin levels and/or activity and their subsequent effect on viral infectivity could offer a possible explanation for the reduced viral titers observed in the lungs of PAR1 antagonist-treated mice, it needs to be confirmed that PAR1 inhibition can reduce the infectivity of other hMPV strains as well. Of interest, replication of several paramyxoviruses is furin-dependent, and this immunomodulatory approach may thus be beneficial not only for hMPV, but for other paramyxovirus infections as well.

In conclusion, we report that PAR1 inhibition, using a non-peptide PAR1 antagonist, is beneficial in hMPV-infected mice. The protective mechanisms conferred by PAR1 antagonists are mediated by a modulation of the innate and adaptive immune responses, and also possibly through a reduction of viral infectivity. Different PAR1 antagonists appear to confer different protection levels during hMPV infection, warranting the evaluation of other PAR1 inhibitors and, importantly, the assessment of delayed treatment on clinical outcome. Importantly, some PAR1 inhibitors are already in clinical trials for other indications, which could accelerate the development of these compounds in the context of acute treatment of viral infections.

## Materials and Methods

### Virus strains and cells

LLC-MK2 cells (ATCC CCL-7) were maintained in minimal essential medium (MEM) (Life Technologies, Carlsbad, CA) supplemented with 10% fetal bovine serum (FBS) (Wisent, Quebec, QC). The hMPV group A strain C-85473, a clinical isolate that was passaged nine to ten times on LLC-MK2, was grown on LLC-MK2 cells in OptiMEM (Life technologies, Carlsbad, CA) supplemented with 0.0002% trypsin (Sigma, St. Louis, MO). Virus stocks were concentrated on Amicon™ columns (Fisher Scientific, Waltham, MA) as previously described [15]. The same protocol was used with 16 flasks of uninfected cells for mock-infected mice.

### Viral titrations

Viral titers were determined by 10-fold serial dilutions of virus in 24-well plates containing LLC-MK2 cells as previously described [10]. Virus titers were reported as 50% tissue culture infectious doses (TCID_50_) per ml. TCID_50_ values were calculated by the Reed and Muench method.

### Compounds

PAR1 agonist (TFLLR-NH_2_) (Genscript, Piscataway, NJ) was reconstituted in H_2_O at a concentration of 10 mM, aliquoted and stored at -20°C. Immediately before intranasal administration, the compound was diluted to 50 µM or 500 µM in OptiMEM. As a control, H_2_O was diluted 1/20 in OptiMEM before administration. PAR1 antagonists (SCH79797 and SCH530348 (Vorapaxar)) (Axon MedChem, Groningen, The Netherlands) were reconstituted at a stock concentration of 22 mM in DMSO and stored at -20°C. Immediately before intranasal administration, these compounds were diluted to 50 µM or 500 µM in OptiMEM. As a control, DMSO was diluted 1/44 in OptiMEM before administration.

### Antiviral activity *in vitro*

In a first experiment, confluent LLC-MK2 cells were incubated for 2 h with 2.5-fold dilutions of PAR1 compounds. Subsequently, cells were washed once with PBS, before infection with 200 TCID_50_ of hMPV. After 5 h, the medium containing the virus was removed and replaced by fresh infection medium. Trypsin was added on days 3 and 5 post infection (pi) and titers were determined on day 6 pi. In a second experiment, hMPV was pre-incubated at 37°C with 2.5-fold dilutions of the compounds for 2 h before being transferred onto confluent monolayers of LLC-MK2 cells. Five hours later, the medium was removed and replaced with fresh infection medium containing the same 2.5-fold dilutions of compounds. Dilutions of compounds were added daily and trypsin was added on days 3 and 5 pi. Titers were then determined on day 6 pi.

### Furin cleavage experiment

HEK293 and COS-1 cells (ATCC CRL-1650) were co-transfected with cDNA from the F protein of the hMPV strain C-85473, tagged with V5 at the C-terminus and cDNA encoding each of the following proprotein convertases (furin, PC5/6, PACE4, PC7 or SKI-1) with/without cDNA encoding hPAR1 (2:1 ratio of hMPV: PC for the double transfection and 2:1:2 ratio of hMPV: PC: hPAR1 for the triple transfection). Twenty hrs post-transfection, cells were treated with hPAR1 agonist, TFLLR-NH_2_ 100 μM, or antagonist, SCH79797 0.1 μM for 4 h, and then incubated for 24 h in fresh serum-free media. Protein lysates from each transfectant were separated on SDS-PAGE followed by Western blot analysis with an anti-V5 mAb as reported [47]. Quantitation was performed using ImageJ software (National Institutes of Health), and normalization was reported to β-actin.

### Quantitative RT-PCR assay for furin transcripts

Lungs were removed on day 5 pi and snap frozen in liquid nitrogen. RNA was extracted using Trizol/chloroform and RNA quality was verified on an agarose gel. cDNA was prepared using 250 ng of total RNA. Quantitative PCR was performed on the MX3005 platform (Stratagen, Santa Clara, CA) using QUANTA Sybergreen (VWR, Radnor, PA). cDNA synthesis and QPCR were performed as previously described [79]. Specific primers sitting on neighboring exons were used for the simultaneous amplification of the normalizing cDNA for ribosomal protein S16 [80].

### BALB/c mouse studies

Four to six-week-old BALB/c mice (Charles River Laboratories) were infected intranasally with 5-8×10^5^ TCID_50_ of hMPV in 25 μl of OptiMEM. As a control, 4–6-week-old BALB/c mice were mock infected. Mice were treated intranasally with 50 or 500 μM of PAR1 agonist (TFLLR-NH_2_) or antagonists (SCH79797 and SCH530348) or with equivalent dilutions of H_2_O or DMSO depending on the compounds. Animals were housed in groups of three to five in micro-isolator cages. The animals were evaluated on a daily basis for weight loss and the presence of clinical signs such as reduced activity and ruffled fur. The humane endpoint was determined at 20% weight loss in accordance with the guidelines provided by the Animal Protection Committee of the Centre Hospitalier Universitaire de Québec. On day 5 pi, six mice per group were euthanized using sodium pentobarbital and lungs were removed for the evaluation of viral titers by cell culture, cytokine levels using a bead-based multiplex immunoassay, histopathological changes using hematoxylin-eosin staining, cell recruitment by flow cytometry, and furin expression levels by quantitative RT-PCR.

### C57Bl/6 mouse studies

Male C57Bl/6 mice (6 weeks old; Charles River Laboratories, Quebec, Canada) were housed in a controlled environment (22°C, 40% humidity, 12: 12 h photoperiods) and had free access to food and water. In a first protocol, after a 12 h fast, under light halothane anaesthesia, a polyethylene catheter was inserted intra-rectally to 3 to 4 cm from the anus. A single administration of TFLLR-NH2 (200 µg/mouse each) or RLLFT-NH2 (200 µg/mouse each) was performed into the distal colon through the catheter, in a volume of 100 µl. Three hours later and under deep anaesthesia (ketamine 60 mg/kg and xylazine 25 mg/kg), mice received an intracolonic infusion of 75 µl of ^51^Cr-EDTA at 2x10^6^ cpm/h for 3 h. Intestinal permeability was assessed by measuring the passage of ^51^Cr-EDTA from the colonic lumen to the blood. Blood was collected by cardiac puncture and was then measured for counts using a gamma counter. In a second protocol, after a 12 h fast, mice received an intraperitoneal injection of the PAR1 antagonist SCH79797 (5mg/kg) or its vehicle (carboxymethyl cellulose), then, one h later, under light halothane anaesthesia, a polyethylene catheter was inserted intra-rectally to 3 to 4 cm from the anus. A single administration of TFLLR-NH2 (200 µg/mouse each) or saline was performed into the distal colon through the catheter, in a volume of 100 µl. Three hours later and under deep anaesthesia (ketamine 60 mg/kg and xylazine 25 mg/kg), mice received an intracolonic infusion of 75 µl of ^51^Cr-EDTA at 2x10^6^ cpm/h for 3 h. Intestinal permeability was assessed by measuring the passage of ^51^Cr-EDTA from the colonic lumen to the blood. Blood was collected by cardiac puncture and was then measured for counts using a gamma counter.

### Ethics statement

The present studies in BALB/c mice were approved by the Animal Protection Committee of the Centre Hospitalier Universitaire de Québec according to the guidelines of the Canadian Council on Animal Care (CPAC 10-082-3). Studies with C57Bl/6 mice were conducted under the standards of the Canadian Council on Animal Care and approved by the Animal Care Committee of the University of Calgary.

### Pulmonary viral titers

Lungs were removed on day 5 pi and snap frozen in liquid nitrogen. The lungs were subsequently weighed, homogenized in 1 ml of PBS and then centrifuged at 2000 rpm for 10 min. The supernatant was used to determine viral titers reported as TCID_50_.

### Pulmonary cytokine levels

An aliquot of 250 µl of lung homogenates was added to 250 µl of 50 mM KPO_4_, pH 6.0 buffer containing 0.2% CHAPS (Sigma, St. Louis, MO) and 0.2% protease inhibitor cocktail (Sigma, St. Louis, MO) and then stored at -20°C. On the day of the experiment, samples were centrifuged at 13,800 × g for 10 min at 4°C and then 50 μl of the supernatant were used for cytokine quantification. Levels of interleukin (IL)-4, IL-6, IL-12(p40), IL-12(p70), IFN-γ, KC, MCP-1, MIP-1α, RANTES were determined using 9-plex mouse bead kits (Millipore, Billerica, MA) according to the manufacturers’ instructions. Experiments were performed in a 96-well filter plate and results were analyzed with the Luminex system (Qiagen, Germantown, MD).

### Histopathology

Lungs were removed on day 5 pi, and fixed with 4% buffered formalin. Fixed lungs were subsequently embedded in paraffin, sectioned in slices of 5 μm, and stained with hematoxylin-eosin. The histopathological scores were determined by two independent researchers who were blinded to experimental data. A semi-quantitative scale was used to score bronchial/endobronchial, peribronchial, perivascular, interstitial, pleural and intra-alveolar inflammation as previously described [10].

### Polychromatic flow cytometry

Lungs were removed on day 5 pi, perfused with 1 ml of endotoxin-free PBS (Cederlane, Burlington, ON) and incubated at 37°C in 1 ml (1 mg/ml) Collagenase D (Roche, Nutley, NJ) in Hank’s Balanced Salt Solution (HBSS) (Wisent, Quebec, QC). After 45 min, 2 U of Dnase I were added and the lungs were incubated at 4°C for another 15 min. Tissues were disrupted mechanically through a 40-μm filter, and red blood cells were lysed using the ACK buffer (Life technologies, Carlsbad, CA). Cells were incubated with FcBlock (BD Biosciences, Franklin Lakes, NJ) and fluorochrome-conjugated antibodies in endotoxin-free PBS containing 1% FBS and 1% HEPES for 20 min at 4°C. After washing, cells were fixed in 2% formaldehyde in endotoxin-free PBS containing actinomycin D (20 µg/ml) (Cederlane, Burlington, ON). Surface marker antibodies were specific for murine CD3, CD4, CD11b, CD11c, Ly6G, I-A/I-E (BD Biosciences, Franklin Lakes, NJ) and F4/80 (Biolegend, San Diego, CA). The 7-AAD dye (20 µg/ml) (Biolegend, San Diego, CA) was used to exclude dead cells. Cells were analyzed using a BD FACSCantoA system and gating of positive cells was based on fluorescence minus one (FMO) controls. All analyses were performed using the FCS express V4 software.

### Statistical analysis

All statistical analyses were performed using the Prism 5 software. Weight loss, pulmonary cytokine/chemokines levels and histopathology scores were analyzed by two-way ANOVA. Lung viral titers and furin expression levels were analyzed using a Student t-test and cell recruitment was analyzed using one-way ANOVA.

## Supporting Information

Figure S1 **Specificity of the PAR1 agonist and PAR1 antagonist in male C57Bl6 mice.** A) Effects of intralumenal administration of the PAR1 agonist (TFLLR-NH_2_) (200 µg) or the inactive control peptide (RLLFT) (200 µg), in the colon of wild-type (PAR1+/+) or PAR1-deficient mice (PAR1-/-) on intestinal permeability: passage of a macromolecule (CrEDTA) from the lumen to the blood, observed 3 h after the intracolonic administration of TFLLR or RLLFT. N=8 per group in each group (** p<0.01). Significantly different from saline or control peptide (RLLFT)-treated group. B) Effects of intralumenal administration of the PAR1 agonist (TFLLR-NH_2_) in the colon of mice that were treated with the PAR1 antagonist (SCH79797) or its vehicle, on intestinal permeability: passage of a macromolecule (CrEDTA) from the lumen to the blood, observed 3 h after the intracolonic administration of TFLLR or RLLFT. N=8 per group in each group. (** p<0.01) significantly different from saline-treated group.
(TIF)

Figure S2 **Weight loss in hMPV-infected mice treated with PAR1 antagonist SCH79797 or SCH530348.** Groups of 6 mice were infected intranasally with hMPV (7 x10^5^ TCID_50_) or mock infected and simultaneously treated for 5 days with a single daily dose of 500 µM of one of two PAR1 antagonists, SCH79797 or SCH530348, then monitored daily for weight loss and mortality during 14 days. The horizontal bar underneath the graphic indicates the timing and duration of treatment. Significant differences in weight loss were observed between SCH79797-treated mice (*) or SCH530348-treated mice (†) and untreated mice based on a two-way ANOVA (** p<0.01, *** p<0.001, † p<0.05). Arrows and numbers indicate the mice that reached the endpoint and were sacrificed (full line: untreated mice, dotted line: SCH530348-treated mice).
(TIF)
