# Supplementary figures and images for: Modulation of Protease Activated Receptor 1 Influences Human Metapneumovirus Disease Severity in a Mouse Model
Source: PLoS One. 2013 Aug 28;8(8):e72529. doi: 10.1371/journal.pone.0072529 (PMC3755973; doi:10.1371/journal.pone.0072529)

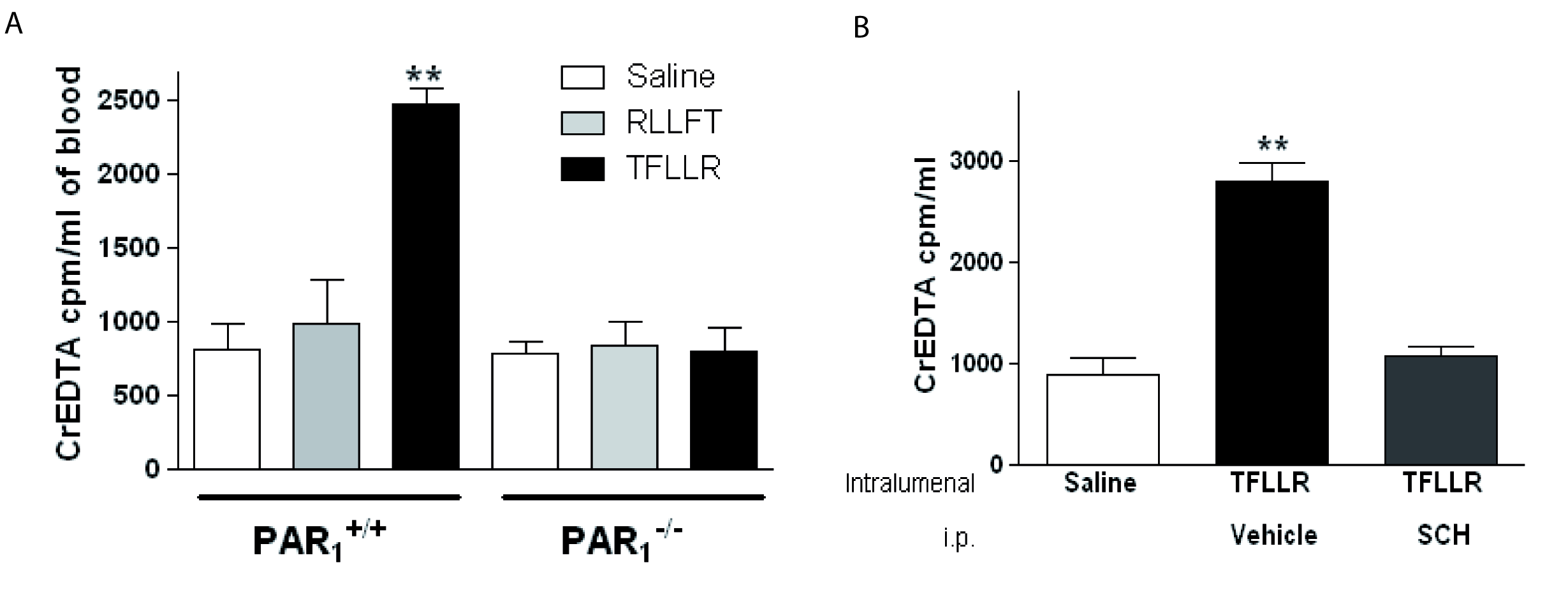

Supplement: Figure S1 — Specificity of the PAR1 agonist and PAR1 antagonist in male C57Bl6 mice. A) Effects of intralumenal administration of the PAR1 agonist (TFLLR-NH2) (200 µg) or the inactive control peptide (RLLFT) (200 µg), in the colon of wild-type (PAR1+/+) or PAR1-deficient mice (PAR1-/-) on intestinal permeability: passage of a macromolecule (CrEDTA) from the lumen to the blood, observed 3 h after the intracolonic administration of TFLLR or RLLFT. N=8 per group in each group (** p<0.01). Significantly different from saline or control peptide (RLLFT)-treated group. B) Effects of intralumenal administration of the PAR1 agonist (TFLLR-NH2) in the colon of mice that were treated with the PAR1 antagonist (SCH79797) or its vehicle, on intestinal permeability: passage of a macromolecule (CrEDTA) from the lumen to the blood, observed 3 h after the intracolonic administration of TFLLR or RLLFT. N=8 per group in each group. (** p<0.01) significantly different from saline-treated group. (TIF) [file pone.0072529.s001.tif]

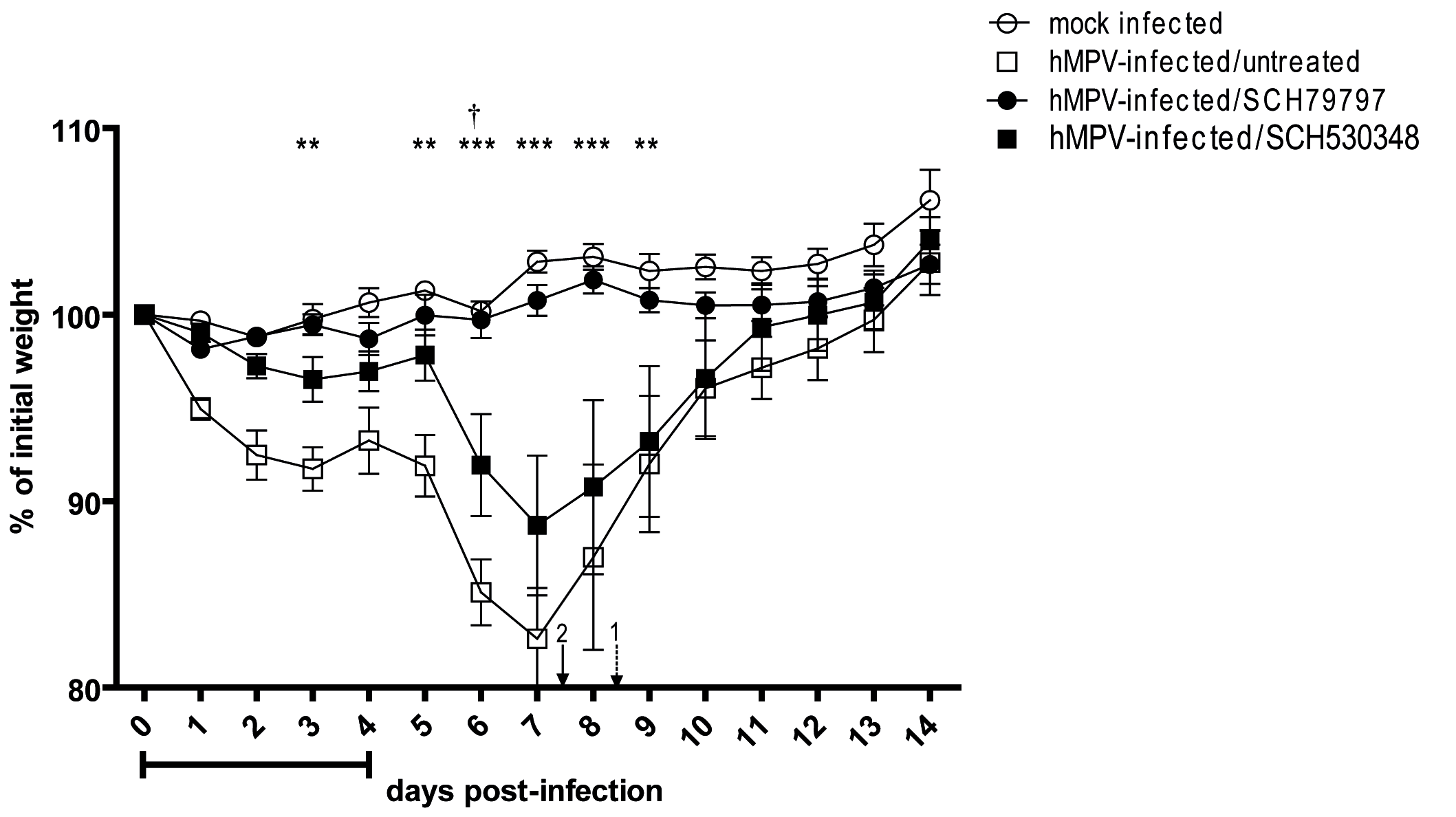

Supplement: Figure S2 — Weight loss in hMPV-infected mice treated with PAR1 antagonist SCH79797 or SCH530348. Groups of 6 mice were infected intranasally with hMPV (7 x10^5^ TCID50) or mock infected and simultaneously treated for 5 days with a single daily dose of 500 µM of one of two PAR1 antagonists, SCH79797 or SCH530348, then monitored daily for weight loss and mortality during 14 days. The horizontal bar underneath the graphic indicates the timing and duration of treatment. Significant differences in weight loss were observed between SCH79797-treated mice (*) or SCH530348-treated mice (†) and untreated mice based on a two-way ANOVA (** p<0.01, *** p<0.001, † p<0.05). Arrows and numbers indicate the mice that reached the endpoint and were sacrificed (full line: untreated mice, dotted line: SCH530348-treated mice). (TIF) [file pone.0072529.s002.tif]
